# Hypertrophic Chondrocytes in the Rabbit Growth Plate Can Proliferate and Differentiate into Osteogenic Cells when Capillary Invasion Is Interposed by a Membrane Filter

**DOI:** 10.1371/journal.pone.0104638

**Published:** 2014-08-14

**Authors:** Tetsuya Enishi, Kiminori Yukata, Mitsuhiko Takahashi, Ryosuke Sato, Koichi Sairyo, Natsuo Yasui

**Affiliations:** Department of Orthopedics, Institute of Health Biosciences, Tokushima University Graduate School, Tokushima, Japan; University of Massachusetts Medical, United States of America

## Abstract

The fate of hypertrophic chondrocytes during endochondral ossification remains controversial. It has long been thought that the calcified cartilage is invaded by blood vessels and that new bone is deposited on the surface of the eroded cartilage by newly arrived cells. The present study was designed to determine whether hypertrophic chondrocytes were destined to die or could survive to participate in new bone formation. In a rabbit experiment, a membrane filter with a pore size of 1 µm was inserted in the middle of the hypertrophic zone of the distal growth plate of ulna. In 33 of 37 animals, vascular invasion was successfully interposed by the membrane filter. During 8 days, the cartilage growth plate was enlarged, making the thickness 3-fold greater than that of the nonoperated control side. Histological examination demonstrated that the hypertrophic zone was exclusively elongated. At the terminal end of the growth plate, hypertrophic chondrocytes extruded from their territorial matrix into the open cavity on the surface of the membrane filter. The progenies of hypertrophic chondrocytes (PHCs) were PCNA positive and caspase-3 negative. In situ hybridization studies demonstrated that PHCs did not express cartilage matrix proteins anymore but expressed bone matrix proteins. Immunohistochemical studies also demonstrated that the new matrix produced by PHCs contained type I collagen, osteonectin, and osteocalcin. Based on these results, we concluded that hypertrophic chondrocytes switched into bone-forming cells after vascular invasion was interposed in the normal growth plate.

## Introduction

Longitudinal bone growth exclusively occurs in the cartilage growth plate by the regulated process of endochondral ossification.[1], [2], [3], [4], [5] Morphologically, growth plate chondrocytes show a columnar arrangement consisting of three characteristic layers: the resting, proliferative, and hypertrophic zones.[4], [6], [7] The hypertrophic chondrocytes generally behave as terminally differentiated cells undergoing degeneration and apoptosis.[4], [8], [9] The territorial matrix of the last chondrocytes is invaded by capillaries and bone marrow-derived chondroclasts/osteoclasts. New bone is formed on the eroded surface of the cartilage matrix by newly arrived osteoblasts. The majority of the bone-forming cells are thought to be differentiated from marrow stromal cells.

Some researchers have suggested that terminal hypertrophic chondrocytes differentiate into osteogenic using organ culture, cell culture, or an *in vivo* avian model.[10], [11], [12], [13], [14], [15] To the best of our knowledge, no study has reported that hypertrophic chondrocytes can differentiate into bone-forming cells in the mammalian growth plate,[4], [8], [16], [17] because it is difficult to determine the origin of each cell at the chondro-osseous junction, where chondrocytes coexist with bone marrow-derived cells.

The present study aimed to clarify the fate of hypertrophic chondrocytes when vascular invasion and cell recruitment were blocked. A membrane filter with a pore size of 1 µm was surgically interposed at the hypertrophic zone of growth plate in rabbit ulna. Migration of bone marrow-derived cells into the growth plate was completely blocked by the filter, whereas the diffusion of humoral factors from metaphysis was maintained. The results demonstrated that hypertrophic chondrocytes did not undergo apoptosis but proliferated into smaller cells within apparently intact lacunae, extruded from the territorial matrix, and eventually differentiated into osteoblast-like cells, producing new bone matrix by themselves.

## Materials and Methods

### Animal model

**Figure 1:**
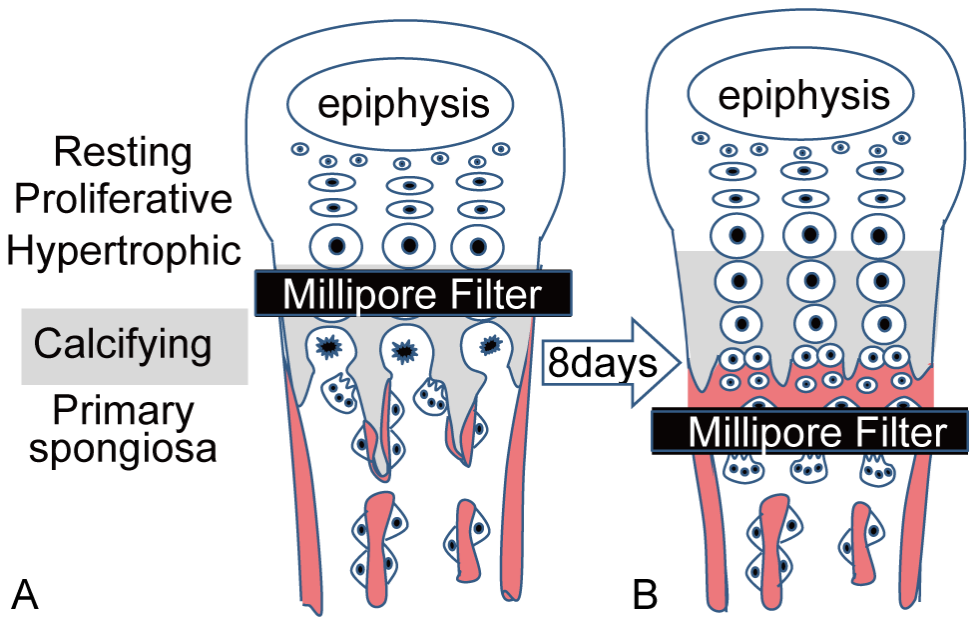
Schematic representation of experimental procedure. The filter was inserted in the middle of the hypertrophic zone and was fixed with a nylon suture to the epiphysis and metaphysis (A). Figure 1B demonstrates the schema of the growth plate after insertion of the membrane filter.

Animal experiments were performed on 37 male Japanese white rabbits (age: 3 weeks, weight: 450–600 g, Kitayama Labes Co., Ltd., Nagano, Japan). The rabbits were housed under standardized environmental conditions and fed standard rabbit chow (RC4, Oriental Yeast Co.). The rabbits were anesthetized with an intravenous injection of ketamine hydrochloride and xylazine at doses of 20 mg/kg and 5 mg/kg body weight, respectively. After exposing the distal growth plate of the left ulna, the hypertrophic zone was transversely cut by a scalpel. Following this, a membrane filter (pore size: 1 µm, mixed cellulose esters, Toyo Roshi Kaisha Ltd., Tokyo, Japan) was interposed in the separated hypertrophic zone and fixed with a nylon suture to the epiphysis and metaphysis (Fig. 1A). This filter protects the growth plate from capillary invasion and cellular recruitments. As a control, the opposite growth plate of the right ulna remained intact. The rabbits were sacrificed by anesthetic overdose. This study was carried out in strict accordance with the recommendations in the Guide for the Care and Use of Laboratory Animals at the University of Tokushima. The protocol was approved by the Committee on the Ethics of Animal Experiments of the University of Tokushima (Permit Number: 07126). All surgery was performed under anesthesia, and all efforts were made to minimize suffering.

### Histology

The distal ulnae were removed and fixed in 4% paraformaldehyde (pH 7.4), decalcified with ethylenediamine tetraacetic acid (EDTA), and embedded in paraffin. Longitudinal 4-µm-thick sections sections were prepared and stained with toluidine blue. Tartrate-resistant acid phosphatase (TRAP) staining was performed as reported previously. [18] The height of the growth plate and the cell size were measured using ImageJ software.

### Immunohistochemistry

After deparaffinization, rehydration, and several washings in PBS, the sections were immersed in methanol, containing 0.3% H_2_O_2_, for 30 min. Enzyme digestion with 1% hyaluronidase (Sigma, St. Louis, MO) was performed at 37°C for 1 h. The sections were then incubated with antibodies for type I collagen (dilution 1∶100, Fuji Chemical Industries Ltd., Toyama, Japan), type II collagen (dilution 1∶100, Fuji Chemical Industries Ltd., Toyama, Japan), type X collagen (dilution 1∶100, gift from Dr. G. J. Gibson, Breech Research Laboratories, Henry Ford Hospital, Detroit, Mich., USA), osteonectin (dilution 1∶100, Takara Shuzo Co Ltd, Kyoto, Japan), osteocalcin (dilution 1∶100, Takara Shuzo Co Ltd, Kyoto, Japan), proliferating cell nuclear antigen (PCNA) (dilution 1∶200, clone PC10, Dakopatts, Copenhagen, Denmark), and cleaved caspase-3 (dilution 1∶100, Biotech Inc., Santa Cruz, CA, USA) overnight at 4°C. After several washings in PBS, the specimens were incubated with the secondary antibodies, a biotinylated antimouse or antigoat IgG (Vector Laboratories, Burlingame, CA, USA), for 1 h. Ab binding was visualized using the Vectastain Avidin–Biotin–Peroxidase Complex Kit (Vector Laboratories) in combination with diaminobenzidine tetrahydrochloride according to the manufacturer's instructions. The sections were counterstained with hematoxylin or methyl green.

### *In situ* hybridization

For the preparation of the probes, the rabbit growth plate was obtained from the distal ulna and immediately frozen in liquid nitrogen and broken down. Total RNAs were then extracted. The reverse transcription reaction was performed at 42°C using oligo-dT. Type I collagen, type II collagen, type X collagen, osteopontin, and osteonectin were then cloned by PCR reaction. The oligonucleotide sequences of the primers used are shown in Table 1. Objective fragments were cloned into pDrive vector (QIAGEN GmbH, Hilden, Germany). The genes were conversed into *Escherichia coli*, amplified, and then linearized and transcribed using T7 and SP6 polymerase into antisense and sense transcripts using the DIG RNA Labeling Kit (Roche Applied Science, Penzberg, Germany). *In situ* hybridization was performed using RNA probes at 50°C for 16 h, and the sections were washed under high stringency. DIG-labeled molecules were detected using NBT/BCIP (Roche Applied Science, Penzberg, Germany) as the substrate for anti-DIG Ab-coupled alkaline phosphatase. The sections were counterstained with methyl green.

**Table 1:** Oligonucleotide primers used in RT-PCR analysis.

Gene	Accession no.	Primer sequence
Col1a1	AY633663.1	Sense: 5′- ACACTGGGGCAACCTGCGTG -3′ Antisense: 5′- GCCTCGGTGGACATGAGGCG -3′
Col2a1	AF050170.1	Sense: 5′- CCCTGTCGGTCCCTCTGGCA -3′ Antisense: 5′- AGGCGCACATGTCGATGCCG -3′
Col10a1	AF247705.1	Sense: 5′- AAGTGGACCGAAAGGAGACA -3′ Antisense: 5′- TGGAAACCCATTCTCACCTC -3′
Osteonectin	AF247647.1	Sense: 5′- GTACCTGTGGGAGCCAACC -3′ Antisense: 5′- CAAACCTTCTCAAACTCGC -3′
Osteocalcin	NM_199173.4	Sense: 5′- CTCCAGGGGATCCGGGTA -3′ Antisense: 5′- AAGCCCAGCGGTGCAGAGT -3′

### Transmission electron microscopy (TEM)

The tissues from the distal ulna were fixed in 2.5% glutaraldehyde and 2% paraformaldehyde in 0.067 M sodium cacodylate with 0.7% ruthenium hexamine trichloride for 18 h, postfixed in 1% osmium tetroxide, and then dehydrated and embedded in Epon 812. Semithin sections were cut at 1 µm and stained with toluidine blue. Ultrathin sections were contrasted with uranyl acetate and lead citrate. Images were obtained using a Hitachi H7650 electron microscope (Hitachi, Tokyo, Japan).

### Image analysis

Immunohistochemistry, *in situ* hybridization, and TEM were performed on several sections until the same staining pattern was observed three times. Cells with diffusely stained cytoplasm or nuclei for PCNA or cleaved caspase-3 were regarded as positive cells.

### Statistical analysis

Statistical analysis was performed using Student's t-test. The results are shown as the mean ± SD. p values<0.05 were considered significantly different. SPSS version-21 software was used for statistical analysis of the data.

## Results

Fig. 1A and 1B summarize the morphological events observed after inserting the membrane filter into the growth plate. In 33 of 37 animals, the membrane filter remained intact after the operation. In those cases, vascular invasion was completely blocked and there was a dramatic increase in the height of the cartilaginous growth plate. The hypertrophic zone of chondrocytes was exclusively elongated and calcified in part. At the terminal end of the elongated cartilage, the hypertrophic chondrocytes escaped from their territorial matrix and switched into two small round cells, producing new bone matrix near the surface of the membrane filter. TRAP-positive multinuclear cells attached to the opposite surface of the filter.

### Radiological findings

**Figure 2:**
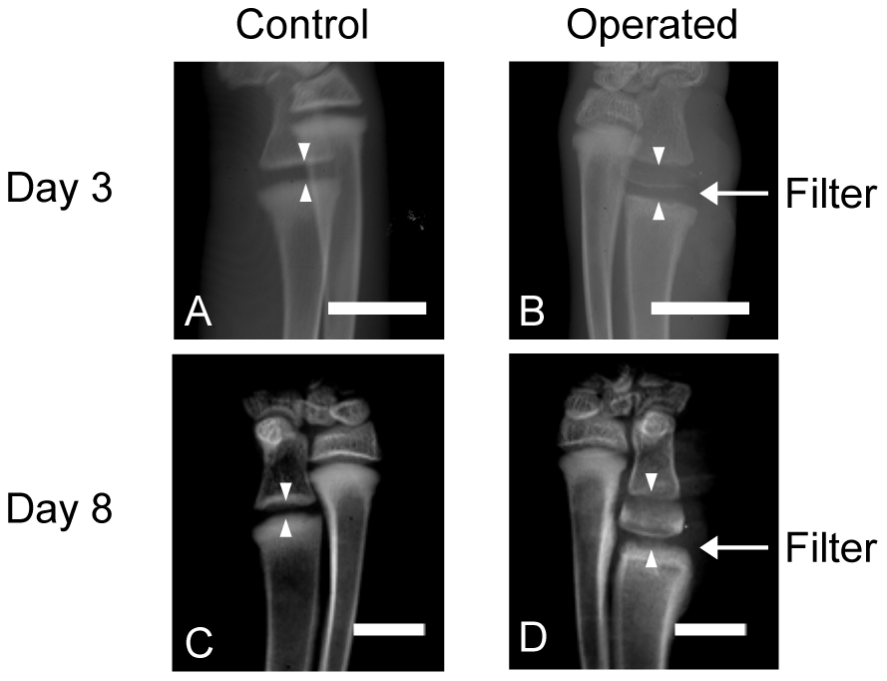
Radiological time course. The height of the growth plate (distance between two white arrow heads) strikingly increased on the operated side. The white arrow indicates the approximate position of the inserted filter. The elongated cartilage was progressively calcified. Bar: 5 mm (A–D).

Fig. 2 shows the radiological time course of the distal growth plate of ulnae after insertion of the membrane filter. By day 3, the total height of the growth plate (distance between two arrow heads in Fig. 2A, 2B) was significantly increased on the operated side. Faint calcification was observed in the elongated growth plate (Fig. 2B). By day 8, the total height of the growth plate had increased even further and the calcification had become more prominent (Fig. 2D). There was no significant difference in the total bone length between the operated side and nonoperated control side.

### Histological changes at the growth plate

**Figure 3:**
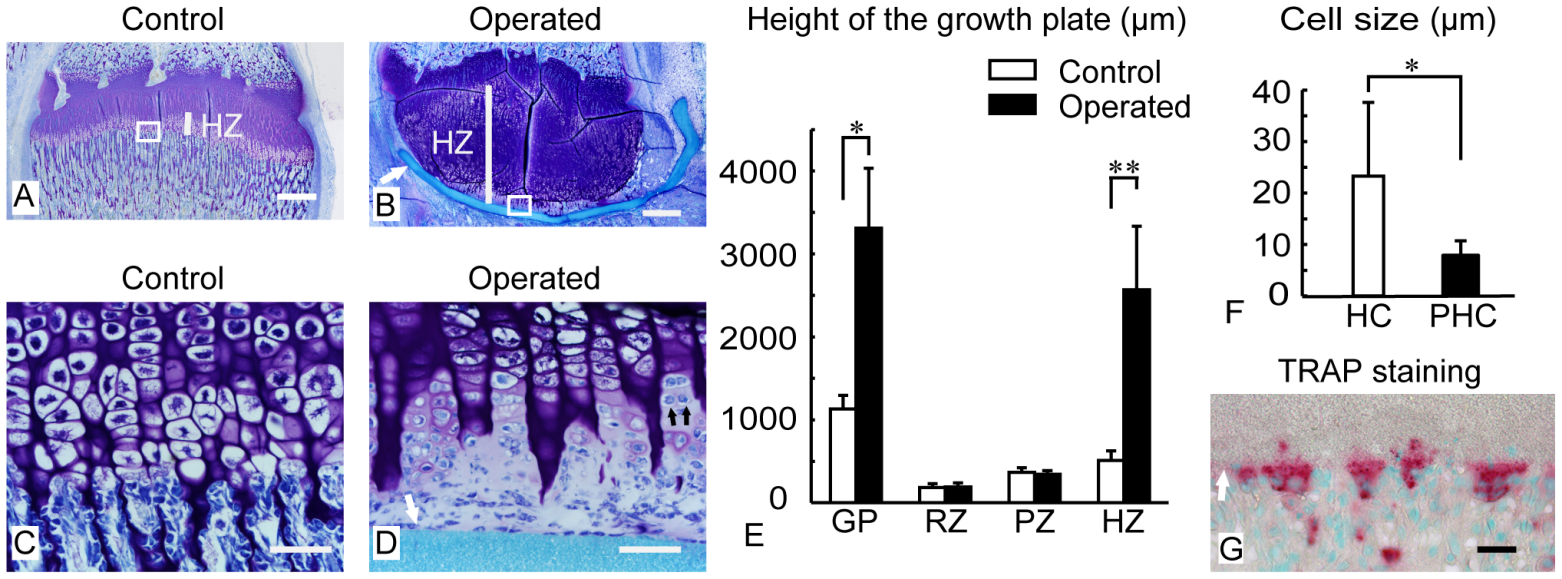
Histological sections of the growth plate with toluidine blue and TRAP staining. The cartilage matrix was metachromatically stained with toluidine blue at the control (A, C) and the operated (B, D) growth plate. There was a striking enlargement of the metachromatic growth cartilage in the operated side (B). The hypertrophic zone (HZ) was exclusively elongated and is indicated with a white longitudinal line (B, F). Figure C shows (white boxed in Fig. A) an enlarged view of the normal chondro-osseous junction. Figure D shows (white boxed in Fig. B) an enlarged view where small cells are located between hypertrophic chondrocytes (HC) and the inserted filter. At the terminal end of the operated growth plate (D), hypertrophic chondrocytes divided into two small round cells and extruded out of the calcified cartilage (black arrows in D). The progeny of hypertrophic chondrocytes (PHCs) produced a new matrix showing a faint metachromasia. PHCs were smaller than hypertrophic chondrocytes in size (F). TRAP staining with methyl green counterstaining showed TRAP-positive multinucleated giant cells aligned on the surface of the inserted filter at the bone marrow side (G). Bar: 500 µm (A, B), 50 µm (C, D, and G). White arrow indicates the inserted filter (B, D, and G). GP: growth plate, RZ: resting zone, PZ: proliferative zone, HZ: hypertrophic zone. HC: hypertrophic chondrocyte. PHC: progeny of hypertrophic chondrocyte. *, **: p<0.001 (Student's t-test).

On the nonoperated control side, the growth plate chondrocytes showed the longitudinal columnar arrangement typically observed in the process of endochondral ossification (Fig. 3A). At the chondro-osseous junction, the hypertrophic chondrocytes within the calcified matrix shrunk and underwent degeneration. There were apparently empty lacunae at the terminal end of the hypertrophic zone (Fig. 3C). The transverse territorial matrix of the terminal chondrocytes was invaded by bone marrow-derived cells, and new bone was deposited on the surface of the eroded cartilage (Fig. 3C). Bony trabeculae in the primary spongiosa contained the core of the cartilage remnant.

On the operated side, the cartilaginous growth plate was strikingly enlarged (Fig. 3B). In case the filter remained intact, the height of the growth plate was threefold higher than that of the control side (Fig. 3E). Among the three distinct layers, the hypertrophic zone was exclusively elongated (Fig 3E). As judged by radiological examination, the distal half of the elongated cartilage was calcified (Fig. 2). Hypertrophic chondrocytes maintained columnar arrangement within the elongated calcified cartilage, and there was no empty lacuna in the operated growth plate. Small cells sequentially aligned with hypertrophic chondrocyte at the terminal end of the calcified cartilage were observed (black arrows in Fig. 3D). Those progenies of hypertrophic chondrocytes (PHCs) were surrounded by the new matrix, clearly different from the pre-existing metachromatic cartilage matrix. PHCs changed cell shapes from small round to flattened or polygonal in the open cavity between the calcified cartilage and implanted filter (Fig 3D). PHCs were smaller in size than hypertrophic chondrocytes (Fig. 3F). Interestingly, on the opposite surface (bone marrow side) of the membrane filter, TRAP-positive multinucleated giant cells aligned (Fig. 3G). These cells may be attracted to the filter surface by a chemokine or humoral factor(s) derived from the growth plate.

In the cases where the filter was ruptured or out of place (in 4 of 37 animals), the bone marrow-derived cells migrated into the filter, and the blood vessels reached to the growth plate. Ordinal processes of endochondral ossification were observed in those cases, and the enlargement of the cartilaginous growth plate did not occur (data not shown).

### TEM

**Figure 4:**
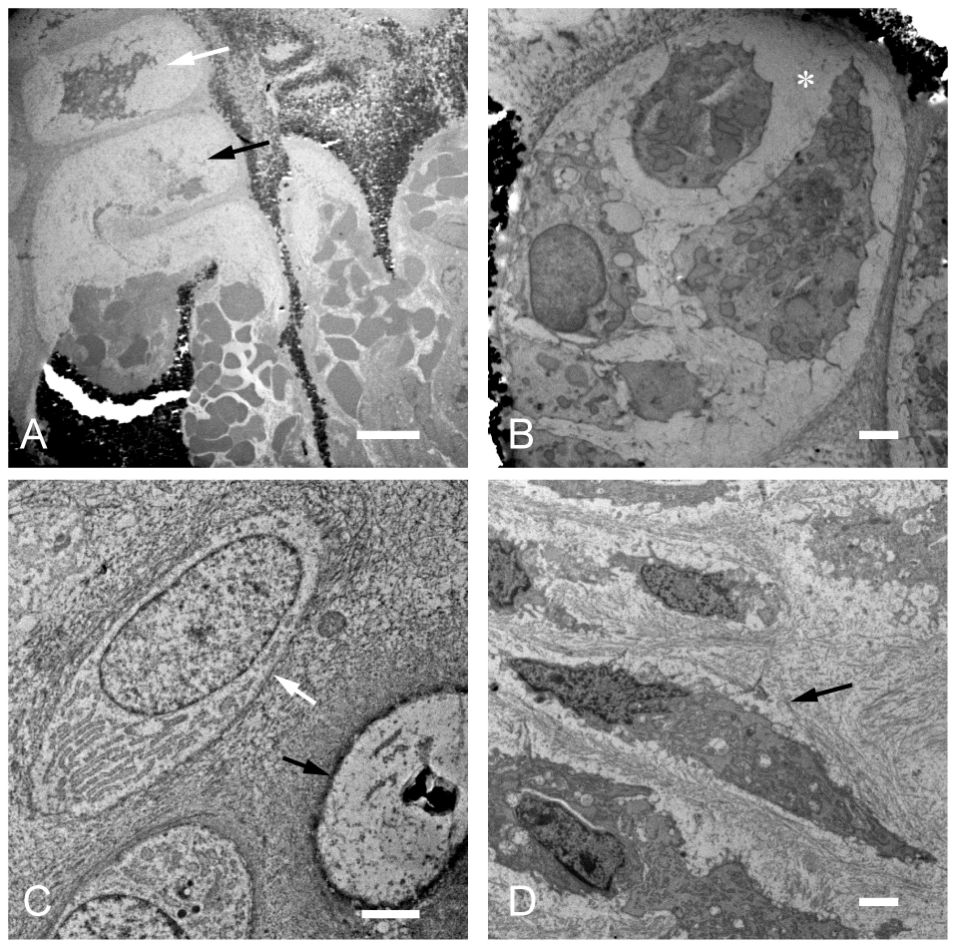
Ultrastructure of the growth plate chondrocytes and PHCs. In the nonoperated control growth plate, a terminally differentiated hypertrophic chondrocyte within an intact lacuna (white arrow in A) showed fragmentation. Open lacunae were filled with red blood cells (A). Longitudinal columns of the cartilage matrix were mineralized (shown as dark particles). In the operated growth plate, an apparently intact lacuna was occupied with multiple cells considered to be PHCs (B). There was no hematocyte invasion at the operated side. PHCs were surrounded by a new homogenous matrix (* in B) containing poor collagen fibers. Small round PHCs (white arrow in C) extruded out of the cartilage possessed an intact nucleus and well-developed cytoplasmic organelle (C). Cells that underwent degeneration and fragmentation were infrequently observed (black arrow in Fig. 4C). Flattened or spindle-shaped PHCs (black arrow in D) possessed an eccentric nucleus, active rough ER, and vesicles (D). These cells were surrounded by an extracellular matrix containing thick collagen fibers with a periodicity. Bar: 100 µm (A), 20 µm (B, C, and D).

Fig. 4A shows the osteo-chondral junction ultrastructure in the nonoperated normal growth plate. The terminal hypertrophic chondrocyte within apparently intact lacunae had undergone degeneration and fragmentation (white arrow in Fig. 4A) and eventually resorbed (dark arrow in Fig. 4A). The transverse territorial matrix was invaded by blood vessel, and the open lacunae were filled with red blood cells.

Fig. 4B shows the terminal end ultrastructure of the elongated cartilage from the operated growth plate. Apparently, intact lacuna was occupied by multiple cells containing an intact nucleus and well-developed organelle. These cells were considered to be PHCs because there was no blood vessel invasion. PHCs produced a new homogenous matrix consisting of a rich amorphous ground substance and poor collagen fibers (* in Fig. 4B). As observed by light microscopy with toluidine blue staining, this matrix showed a weak metachromasia clearly distinguishable from the old cartilage matrix (Fig. 3D). The matrix resembled the chondroid bone, an intermediate tissue between the bone and cartilage. [19]

Fig. 4C shows the ultrastructure of small round PHCs extruded out of the calcified cartilage (white arrow in Fig. 4C). The small round cells possessed an intact nucleus and well-developed cytoplasmic organelle. The extracellular matrix consisted of relatively thick bundles of collagen fiber, not usually observed in the cartilage matrix. There were a few neighbor cells that appeared to have undergone degeneration and fragmentation (black arrow in Fig. 4C).

Fig. 4D shows flattened or spindle PHCs near the surface of the implanted membrane filter. These cells had a dark eccentric nucleus and well-developed cytoplasmic organelle (black arrow in Fig. 4D). The matrix of flattened or spindle PHCs predominantly consisted of thick bundles of collagen fibers having a periodicity typically observed in the new bone matrix.

### PCNA and caspase-3 staining

**Figure 5:**
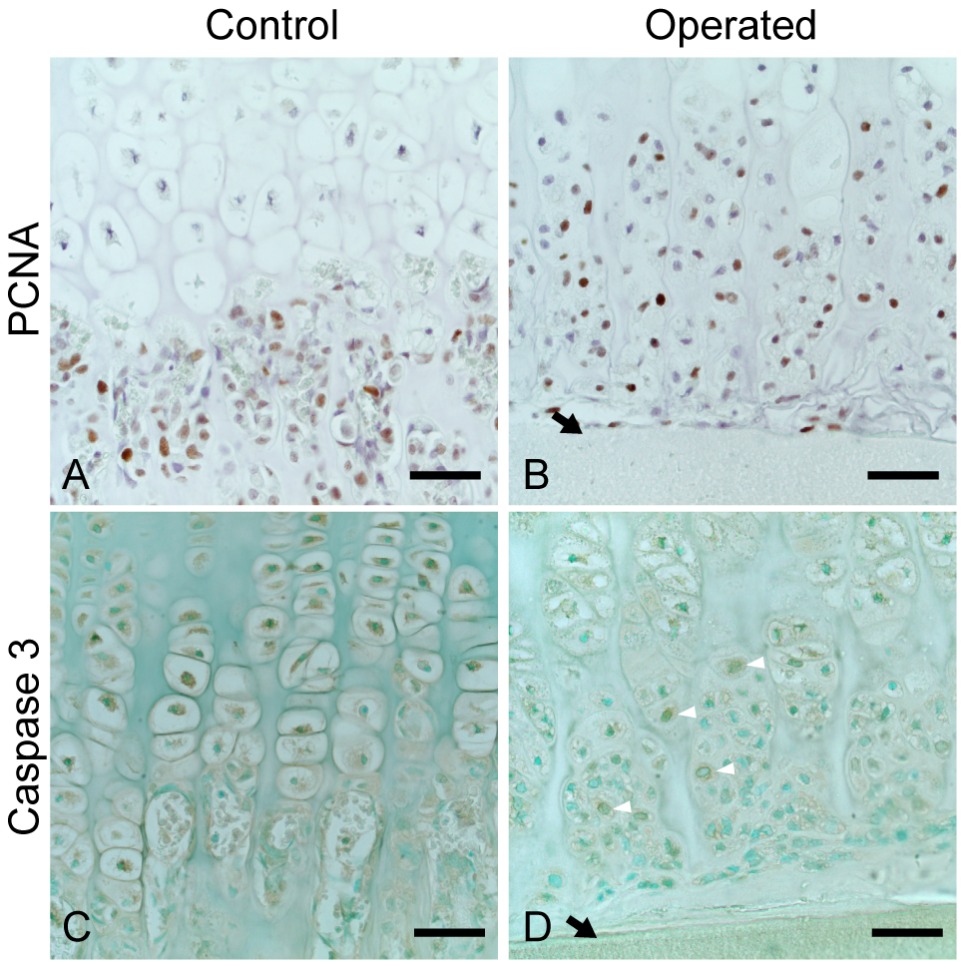
PCNA and caspase-3 staining. Hypertrophic chondrocytes in the control growth plate did not react to PCNA staining (A), while many PHCs in the operated growth plate were PCNA positive (B). In contrast, most hypertrophic chondrocytes of the control growth plate were stained with caspase-3 antibody (C), whereas polygonal and spindle shaped PHCs were negative (D). A few small PHCs were positive against caspase-3 antibody (indicated with white arrow heads). Bar: 50 µm (A–D). PCNA; proliferating cell nuclear antigen.

In the control growth plate, hypertrophic chondrocytes did not react to PCNA staining, while many bone marrow-derived cells positively reacted to PCNA staining (Fig 5A). In the operated side as well, most hypertrophic chondrocytes in the elongated cartilage did not react to PCNA staining. However, the chondrocytes in the terminal few layers of the elongated cartilage reacted to PCNA staining. In addition, many PHCs with different shapes were positive to PCNA staining (Fig. 5B).

Immunostaining with anticleaved caspase-3 antibodies demonstrated that the hypertrophic chondrocytes in the nonoperated intact growth plate were positive to this staining (Fig. 5C). In contrast, in the operated growth plate, none of the hypertrophic chondrocytes of the elongated cartilage reacted to anticleaved caspase-3 antibodies (Fig. 5D). Although some small round PHCs were caspase-3 positive (white arrows in Fig. 5D), polygonal and spindle-shaped PHCs were negative to these antibodies. These results were consistent with the ultrastructure demonstrated by TEM (Fig. 4).

### Collagen phenotypes of PHCs

**Figure 6:**
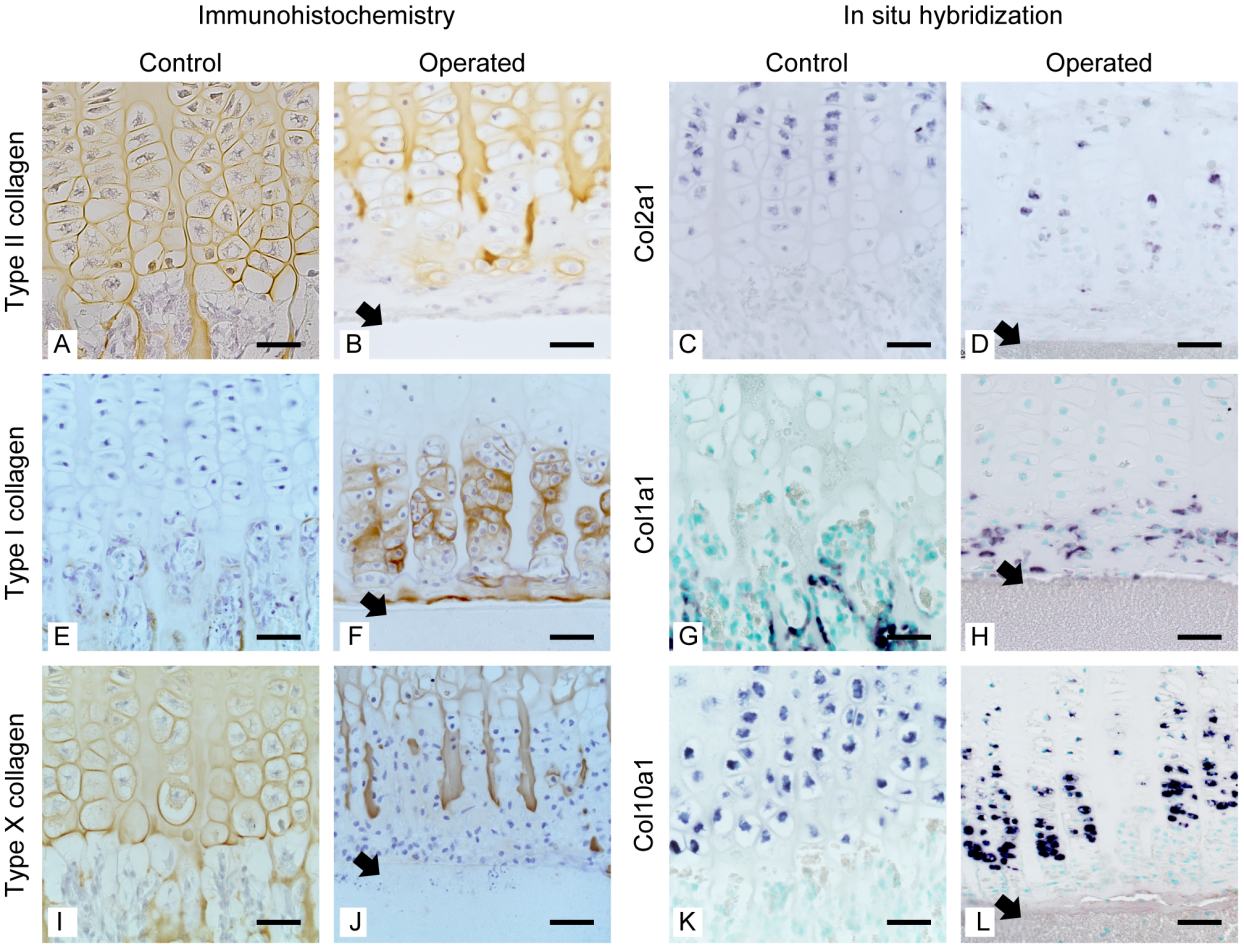
Expression of type I, II, and X collagen. Coronal section of the distal growth plate at the chondro-osseous junction (boxed in Fig. 3A and 3B). Type II and type X collagens were detected at the normal growth plate (A and I). In contrast, these proteins were not detected at the extracellular matrix of PHCs. However, a few PHCs were surrounded with type II and type X collagen (B, J). The pre-existing cartilage matrix did not react to antibodies against type I collagen in the control (E) and operated (F) growth plate. New bone trabeculae in the primary spongiosa consisted of type I collagen (E). The matrix produced by late hypertrophic chondrocytes and the chondroid bone produced by PHCs in the operated growth plate strongly reacted to antibodies against type I collagen (F). The longitudinal column of old cartilages consisted of type II and type X collagens (B, F, and J). Gene expression detected by *in situ* hybridization was consistent with immunostaining data. Bar: 50 µm (A–L). Black arrows indicate the inserted filter (B, D, F, H, J, and L)

Fig. 6 shows the immunohistochemical localization and gene expression of type I, II, and X collagens at the distal end of the growth plate in sequential histological sections. All cartilaginous matrixes were homogenously stained with antitype II collagen antibodies, both in the control and the operated growth plate (Fig. 6A, 6B). In the primary spongiosa of the control side, the new bone deposited on the surface of the cartilage remnant contained type I collagen (Fig 6E), while the cartilage core itself consisted of type II collagen (Fig 6A). Longitudinal columns of the cartilage matrix did not contain type I collagen (Fig 6E, 6F). Strikingly, the chondroid matrix surrounding PHCs in the operated growth plate contained type I collagen (Fig. 6F). Type X collagen was localized at the calcified cartilage matrix in the control and the operated growth plate (Fig. 6I, 6J). Collagen gene expression detected by *in situ* hybridization was consistent with immunohistological data, although there was some time lag between the two data (Fig.6C, 6D, 6G, 6H, 6K, and 6L).

### Osteonectin and osteocalcin

**Figure 7:**
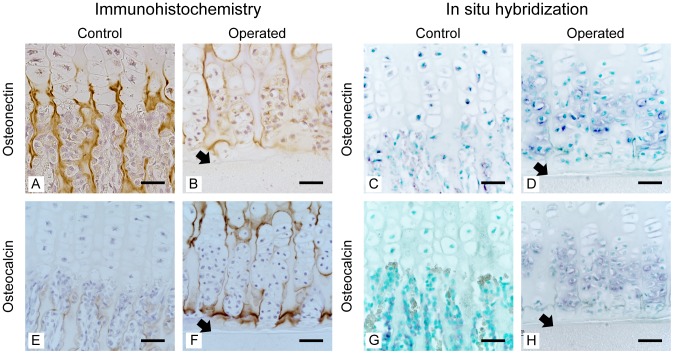
Expression of osteonectin and osteocalcin. Immunolocalization of osteonectin (A, B) and osteocalcin (C, D) was similar to that of type I collagen. New bone and chondroid bone, which were produced by PHCs, contained these proteins (B, F). PHCs in the operated side expressed these genes (D, H). Black arrows indicate the inserted filter (B, F, D, and H). Bar: 50 µm (A–H).

In the control growth plate, osteonectin and osteocalcin were predominantly detected in the new bone matrix of primary spongiosa (Fig 7A, 7E). In the operated growth plate, the new matrix produced by PHCs contained both osteonectin and osteocalcin (Fig 7B, 7F). *In situ* hybridization demonstrated that PHCs expressed both osteonectin and osteocalcin (Fig 7D, 7H). These data suggested that the matrix produced by PHCs was bone.

## Discussion

The fate of hypertrophic chondrocytes during endochondral ossification has been controversial in the literature.[4], [5], [8], [17], [20] Most authors thought that hypertrophic chondrocytes are terminally differentiated cells undergoing degeneration and that new bone is formed by newly arrived cells.[2], [4], [21] However, several authors have reported the possibility of osteogenic differentiation of chondrocytes using organ culture or an *in vivo* system.[11], [13], [15], [16], [22], [23], [24] Particularly, Shimomura et al. reported that isolated chondrocytes from rat growth cartilage had osteogenic potential when they were placed within the Millipore diffusion chamber and implanted into the abdominal cavity.[23] They also described that growth cartilage cells alone did not form new bone and the participation of certain host cells was necessary to initiate osteogenic differentiation.

The present study demonstrated that hypertrophic chondrocytes of the rabbit growth plate could survive, proliferate, and switch into bone-forming cells when vascular invasion was inhibited by a membrane filter. Some PHCs temporarily expressed type II and X collagen; however, they simultaneously expressed osteocalcin, osteonectin, and collagen types I. Beresford described the presence of the chondroid bone as an intermediate tissue between the cartilage and bone.[19] The cells within the chondroid bone resembled chondrocytes in shape and size; however, the matrix was more bone-like.[19], [25] The chondroid bone may occur when and where the cartilage directly turns into the bone.[25] In the present study, the new matrix of PHCs within an intact lacuna was homogenous, amorphous, and colloidal. This matrix showed weak metachromasia by toluidine blue staining. The matrix of flattened or polygonal PHCs did not show metachromasia but contained various bone matrix proteins. The intermediate tissue with a bony matrix and chondrocyte-like cells could be considered as the chondroid bone.

Hypertrophic chondrocytes isolated within intact lacunae do not usually proliferate but undergo degeneration. [4], [9], [26] Electron microscopy data in the present study demonstrate that there were multiple cells within an apparently intact lacuna at the terminal end of the elongated calcified cartilage. We hypothesize that the degeneration and death of hypertrophic chondrocytes are not necessarily a programmed event; however, they are induced by vascular invasion and/or cells from the bone marrow area. When blood supply was interrupted for some reasons, hypertrophic chondrocytes could begin to proliferate and switch into bone-forming cells by themselves. During bone lengthening by distraction osteogenesis, for example, trans-chondroid bone formation was observed for a certain period after osteotomy until the interrupted blood supply recovered. [25]

Roach et al. described that the osteogenic differentiation of hypertrophic chondrocytes involved asymmetric cell divisions and apoptosis.[16], [27], [28] In the present study, PHCs that escaped from the calcified cartilage matrix changed the cell shape from round to polygonal and eventually spindle. Most of them maintained proliferative activity, while a few cells appeared to undergo degeneration and fragmentation. It is however unknown whether this phenomenon was accompanied by asymmetric cell division.

Chondroclast is a multinucleated giant cell that participates in cartilage resorption.[29] The cell is TRAP-positive and morphologically indistinguishable from the osteoclast.[30] The chondroclast/osteoclast is derived from bone marrow stem cells. In the present experiment, TRAP-positive osteoclast-like cells aligned on the surface of the filter at the bone marrow side. This phenomenon was only observed when the filter remained intact. It is interesting to speculate that TRAP-positive cells were attracted to the membrane surface by a certain chemokine or a humoral factor released from the growth plate chondrocytes or PHCs. Any small molecule, but no cell, could pass through the intact membrane filter. Direct contact of chondroclasts/osteoclasts with the calcified cartilage may be crucial to provide ordinary endochondral ossification.

The limitation of this study was the absence of cell lineage tracing. The cellular origin of PHCs is demonstrated by the histology of the preserved specimens. First, sequential arrangements of cells are a necessary but not sufficient condition for the certification of the cellular origin. Second, the serial sections confirmed the gradual transition from hypertrophic chondrocytes to osteoblast-like cells, and no entry of different cells into the growth plate through the membrane filter was observed. Third, hypertrophic chondrocytes adhere to the membrane filter at the rim of the growth plate (Figure S1A, 1B). Therefore, the migration of different cells into the growth plate is not observed.

Another possibility is that cells in the perichondrium are candidate ancestors of these osteogenic cells.[31] In the present study, those cells were not blocked and may have been able to participate in bone formation. However, in our experiment, it was unlikely for those cells to pass through the growth plate or the gap between the inserted filter and growth plate, to attach below typical hypertrophic chondrocytes (indicated by black arrows in Figure 3D), and subsequently to differentiate into osteogenic cells during the experimental period. In addition, we were unable to detect the migration of the skeletal progenitor cells from the perichondrium into the site of osteogenic differentiation via the growth plate in histological examinations, although bone formation by perichondrium reaction for surgical intervention was observed on the surface of the growth plate (Figure S1A–C). At the rim of the terminal end of the elongated growth plate, hypertrophic chondrocytes attached to the filter and maintained their phenotype. There were no skeletal progenitor cells from the perichondrium available for migration into the site of osteogenic differentiation. These findings are also consistent with the fact that these small cells, which are designated as PHCs, are derived from hypertrophic chondrocytes.

In previous researches, lineage tracing using transgenic mice were performed for revealing the fate of hypertrophic chondrocytes and for understanding bone development.[32], [33] Maye et al. examined the origin of primary spongiosa by intercrossing *Col10a1-mCherry* transgenic mice with osteoblast reporter mice.[33] Maes et al. followed the fates of stage-selective subsets of osteoblast lineage cells using tamoxifen-inducible transgenic mice bred to Rosa26R-LacZ reporter mice.[32] These excellent studies were unable to prove that hypertrophic chondrocytes undergo differentiation to osteoblasts, because these experiments also had some limitations.[32], [33], [34] However, the possibility that hypertrophic chondrocytes differentiate to osteoblast was not excluded.[5], [32], [33] Future studies were needed to address the fate of hypertrophic chondrocytes.

The possibility that surgical intervention affects the conversion of hypertrophic chondrocytes into osteoblast-like cells remains to be explored. According to a previous study, a disruption of the normal association with neighboring cells may stimulate the release of active factors, which induce adjacent cells to differentiate.[35] However, without the prevention of cellular and vascular invasion by a filter, osteogenic differentiation was never observed in the incised growth plate in our preliminary study (Figure S2). Salter et al. inserted a sheet of teflon into the hypertrophic zone of the growth plate to completely separate the growth plate from the metaphysis. Subsequently, the growth plate was strikingly elongated; however, osteogenic differentiation of hypertrophic chondrocytes was not observed during their experimental period. [36] Furthermore, the described conversion was not reported in previous *in vivo* studies on the fracture of the growth plate. [36], [37], [38], [39] Therefore, we conclude that surgical intervention does not affect the described differentiation of hypertrophic chondrocytes.

The results described in this research provide some insights into the differentiation of hypertrophic chondrocytes in the growth plate. Future studies are required to reveal the osteogenic differentiation of hypertrophic chondrocytes and may lead to a deeper understanding of the normal mechanisms of skeletal development.

## Conclusions

Our study provides the first report revealing chondrocyte differentiation into osteoblast-like cells in the mammalian growth plate.

## Supporting Information

Figure S1 **Histological sections stained with toluidine blue were shown at the left side of the elongated growth plate (A), at the right side of the elongated growth plate (B), and at the part located slightly above in the Figure S1B (C).** Apparently normal hypertrophic chondrocytes attached to the membrane filter (A, B). White arrowhead indicates the site of the osteogenic differentiation of hypertrophic chondrocytes. There were no migrating cells between the growth plate and the filter. The black arrow indicates the formation of bone by perichondrium reaction (C). The white arrow indicates the inserted membrane filter. Bar: 100 µm (A–C).
(TIF)

Figure S2 **The same surgical intervention was added at the hypertrophic zone of the rabbit growth plate, but a membrane filter was not inserted.** Histological sections stained with toluidine blue demonstrated at the center of the injured growth plate (A) and at the rim of the injured growth plate (B). The line of the chondro-osseous junction was disturbed. The morphological changes shown in Figure 3D were not observed. Higher magnification of Figure S2A shows that osteocalcin was detected at the primary spongiosa, as assessed by immunoshistochemistry (C). Bar: 100 µm (A and B), 50 µm (C). Counterstained with hematoxylin and eosin (C).
(TIF)
