# Supplementary figures and images for: Hypertrophic Chondrocytes in the Rabbit Growth Plate Can Proliferate and Differentiate into Osteogenic Cells when Capillary Invasion Is Interposed by a Membrane Filter
Source: PLoS One. 2014 Aug 14;9(8):e104638. doi: 10.1371/journal.pone.0104638 (PMC4133260; doi:10.1371/journal.pone.0104638)

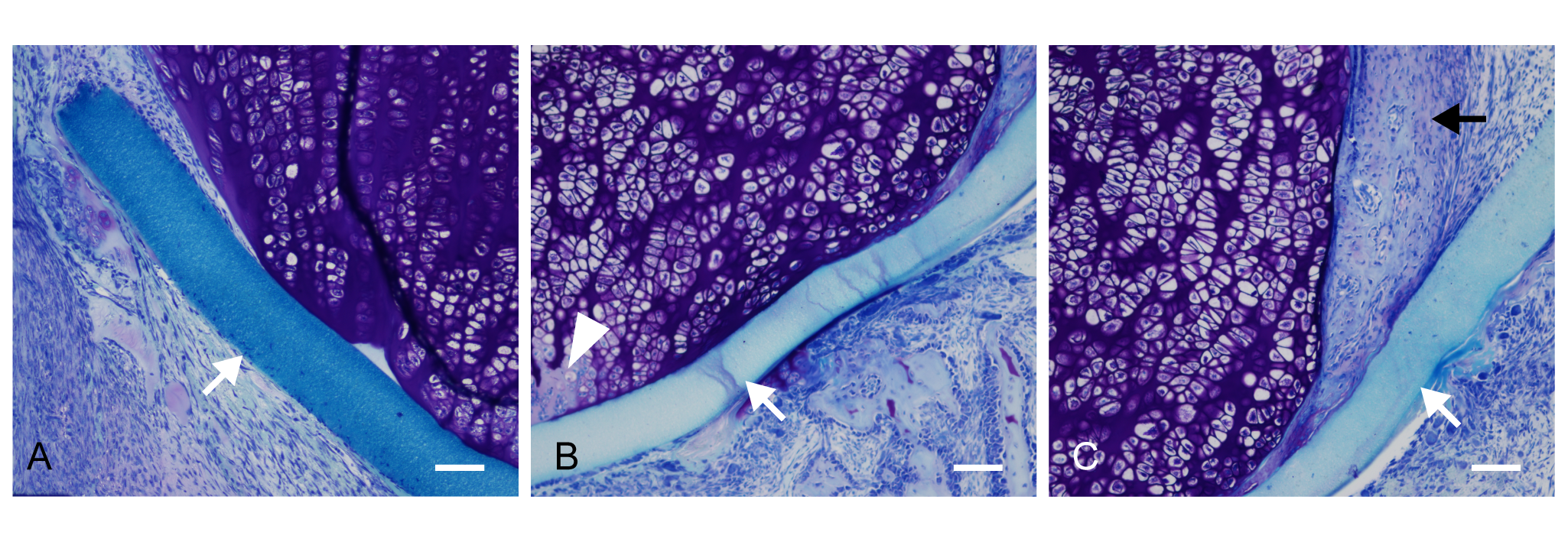

Supplement: Figure S1 — Histological sections stained with toluidine blue were shown at the left side of the elongated growth plate (A), at the right side of the elongated growth plate (B), and at the part located slightly above in the Figure S1B (C). Apparently normal hypertrophic chondrocytes attached to the membrane filter (A, B). White arrowhead indicates the site of the osteogenic differentiation of hypertrophic chondrocytes. There were no migrating cells between the growth plate and the filter. The black arrow indicates the formation of bone by perichondrium reaction (C). The white arrow indicates the inserted membrane filter. Bar: 100 µm (A–C). (TIF) [file pone.0104638.s001.tif]

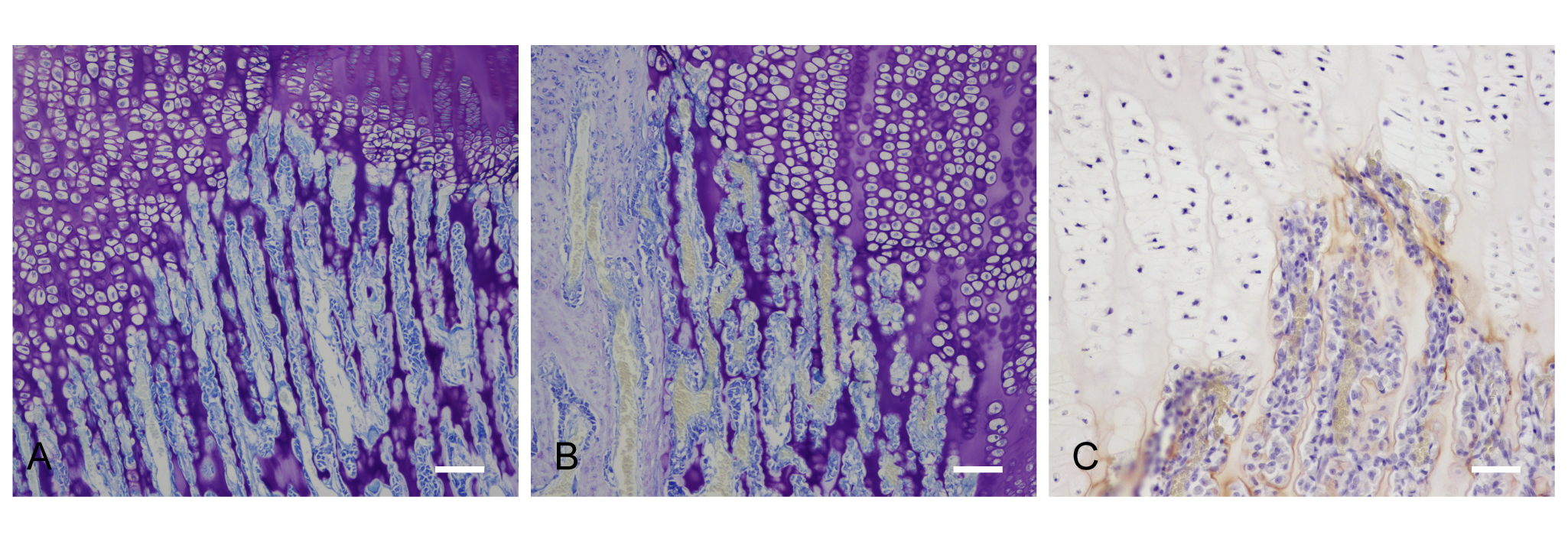

Supplement: Figure S2 — The same surgical intervention was added at the hypertrophic zone of the rabbit growth plate, but a membrane filter was not inserted. Histological sections stained with toluidine blue demonstrated at the center of the injured growth plate (A) and at the rim of the injured growth plate (B). The line of the chondro-osseous junction was disturbed. The morphological changes shown in Figure 3D were not observed. Higher magnification of Figure S2A shows that osteocalcin was detected at the primary spongiosa, as assessed by immunoshistochemistry (C). Bar: 100 µm (A and B), 50 µm (C). Counterstained with hematoxylin and eosin (C). (TIF) [file pone.0104638.s002.tif]
